# Quantifying the evidence for co-benefits between species conservation and climate change mitigation in giant panda habitats

**DOI:** 10.1038/s41598-017-12843-0

**Published:** 2017-10-05

**Authors:** Renqiang Li, Ming Xu, Ryan Powers, Fen Zhao, Walter Jetz, Hui Wen, Qingkai Sheng

**Affiliations:** 1 Key Laboratory of Ecosystem Network Observation and Modeling, Institute of Geographic Sciences and Natural Resources, the Chinese Academy of Sciences, 11A Datun Road, Beijing, 100101 China; 2 Department of Ecology, Evolution and Natural Resources, Rutgers University, New Brunswick, NJ 08901 USA; 3 Department of Ecology and Evolutionary Biology, Yale University, 165 Prospect Street, New Haven, 06520 CT USA; 4 College of Urban and Environmental Science, Peking University, 5 Yiheyuan Road, Beijing, 100871 China

## Abstract

Conservationists strive for practical, cost-effective management solutions to forest-based species conservation and climate change mitigation. However, this is compromised by insufficient information about the effectiveness of protected areas in increasing carbon storage, and the co-benefits of species and carbon conservation remain poorly understood. Here, we present the first rigorous quantitative assessment of the roles of giant panda nature reserves (NRs) in carbon sequestration, and explore the co-benefits of habitat conservation and climate change mitigation. Results show that more than 90% of the studied panda NRs are effective in increasing carbon storage, with the mean biomass carbon density of the whole NRs exhibiting a 4.2% higher growth rate compared with lands not declared as NRs over the period 1988–2012, while this effectiveness in carbon storage masks important patterns of spatial heterogeneity across the giant panda habitats. Moreover, the significant associations have been identified between biomass carbon density and panda’s habitat suitability in ~85% NRs and at the NR level. These findings suggest that the planning for carbon and species conservation co-benefits would enhance the greatest return on limited conservation investments, which is a critical need for the giant panda after its conservation status has been downgraded from “endangered” to “vulnerable”.

## Introduction

Many conservation efforts attempt to develop win–win strategies that would be highly efficient at both mitigating climate change and protecting biodiversity^1–5^. The second largest source of greenhouse gas emissions in the world is from deforestation and forest degradation^6^. PAs as all public areas under land-use restrictions contribute to protecting forest ecosystems, and serve as vital tool for protecting biodiversity and mitigating climate change through reducing deforestation and forest degradation, and promoting reforestation^7–10^. Contemporary conservation management increasingly focuses on preserving both threatened species habitats and maximizing carbon storage in PAs^11,12^. Therefore, a scientifically sound conservation management plan requires a better understanding of the role and effectiveness of PAs in carbon storage and the relationship between species conservation and carbon stocks^13,14^. To date, some progress has been made on quantifying PAs’ effectiveness in reducing deforestation in tropical forest ecosystems^15–18^ and securing co-benefits between biodiversity conservation and carbon stocks at the global scale^19–21^. But information about the implications of such studies in the past is very limited. Without an evidence base for this conservation effectiveness in the species and carbon co-benefits, it is very difficult for decision makers to design effective policies and programs^22,23^.

**Figure 1:**
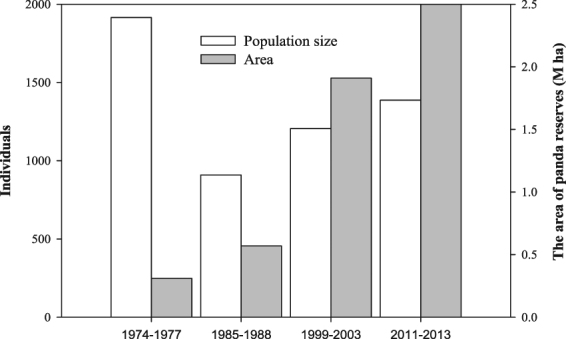
Estimated area of panda nature reserves and population size of wild giant pandas in Sichuan from the previous four National Giant Panda Surveys (Survey period -I: 1974–1977, II: 1985–1988, III: 1999–2003, IV: 2011–2013).

The giant panda (*Ailuropoda melanoleuca*), a universal symbol of wildlife conservation, was once widely distributed across about one quarter of China, but its current distributions are restricted to about 1% of the historical distributional range in China^24–27^. An extended period of habitat destruction, human disturbance, and climate change have all contributed to restricting today’s pandas to six isolated mountain ranges in Sichuan, Shaanxi and Gansu provinces^28–31^. Five of the six mountain ranges that still have wild giant pandas are located in Sichuan Province, which is home to more than 70% of the world’s total wild population^32^. In these ranges, 46 nature reserves (NRs) have been designated to protect this species and its habitats, and four large-scale surveys (National Giant Panda Survey) have been carried out since 1970s to monitor their recovery. The results of these surveys indicated that both the wild giant panda population and their habitat range have greatly increased between 1980 and 2013. The population has steadily recovered from 909 to 1387 (52% increase), and the total area of nature reserves has expanded from 0.57 to 2.53 million ha (344% increase) in Sichuan Province (Fig. 1). The direct measures of changes in the provision of habitats for giant panda show that conservation efforts and forest restoration in the past decades have also greatly improved the quality and area of panda habitats^33^. Despite these great conservation successes^31^, it is unclear as to whether the current management plan can concurrently achieve both giant panda conservation and climate change mitigation, or whether these conservation policies need to be revised to take into account the escalating challenges of climate change mitigation and potential climate change-induced habitat shifts to non-reserve areas^34–37^ to enhance the greatest return on limited conservation investments, especially after the International Union for Conservation of Nature (IUCN) officially downgraded its conservation status from “endangered” to “vulnerable”, which may lead to a lower security for pandas, mainly because the potential conservation investments would be reduced.

Here we provide the first detailed analysis of the role the giant panda NRs play in carbon sequestration, and the relationship between giant panda habitat quality and biomass carbon density in NRs in the Sichuan Province. We produced spatially explicit maps of ecosystem biomass carbon stocks and land cover for 1988 and 2012 based on forest inventory data and field investigation, and then we adopted the matching method to compare the changes in carbon density inside and outside NRs over this period. Lastly, we analyzed the relationship between habitat suitability and biomass carbon density to explore the potential co-benefits of species and carbon conservation.

## Results

**Figure 2:**
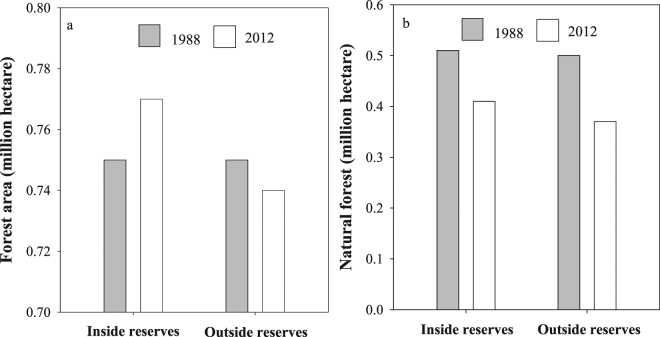
Changes in forest area inside panda reserves and outside of reserves in Sichuan Province. (**a**) The area of all forests (including natural forest and non-natural forest) is shown for 1988 and 2012. (**b**) The area of natural forest for 1988 and 2012.

In 1988 more than 50% of these studied NRs were covered by all forests (both non-natural and natural forests) (0.75 M ha). Non-natural forests include secondary forests and plantation in this area. By 2012, forests in the NRs generally expanded (0.02 M ha, 3.1% increase), while forests outside of NRs with the most similar land characteristics suffered forest loss (0.01 M ha, 1.3% loss) (Fig. 2a). Specifically, forests in the NRs experienced 0.12 M ha of conversion or transition to another land cover, and 0.14 M ha of reforestation, whereas forests outside of NRs outside of NRs experienced 0.14 M ha of conversion and 0.13 M ha of reforestation. We also estimated the effect of protection on natural forest during this period to permit more accurate conservation impact estimates. The area of natural forest in NRs decreased from 0.51 M ha to 0.41 M ha, while it dropped from 0.50 M ha to 0.37 M ha in non-reserve lands (Fig. 2b). Our results indicated that despite their status as NR, natural forest loss still occurred, but that this loss in panda NRs was reduced compared to lands not declared as reserves.

**Figure 3:**
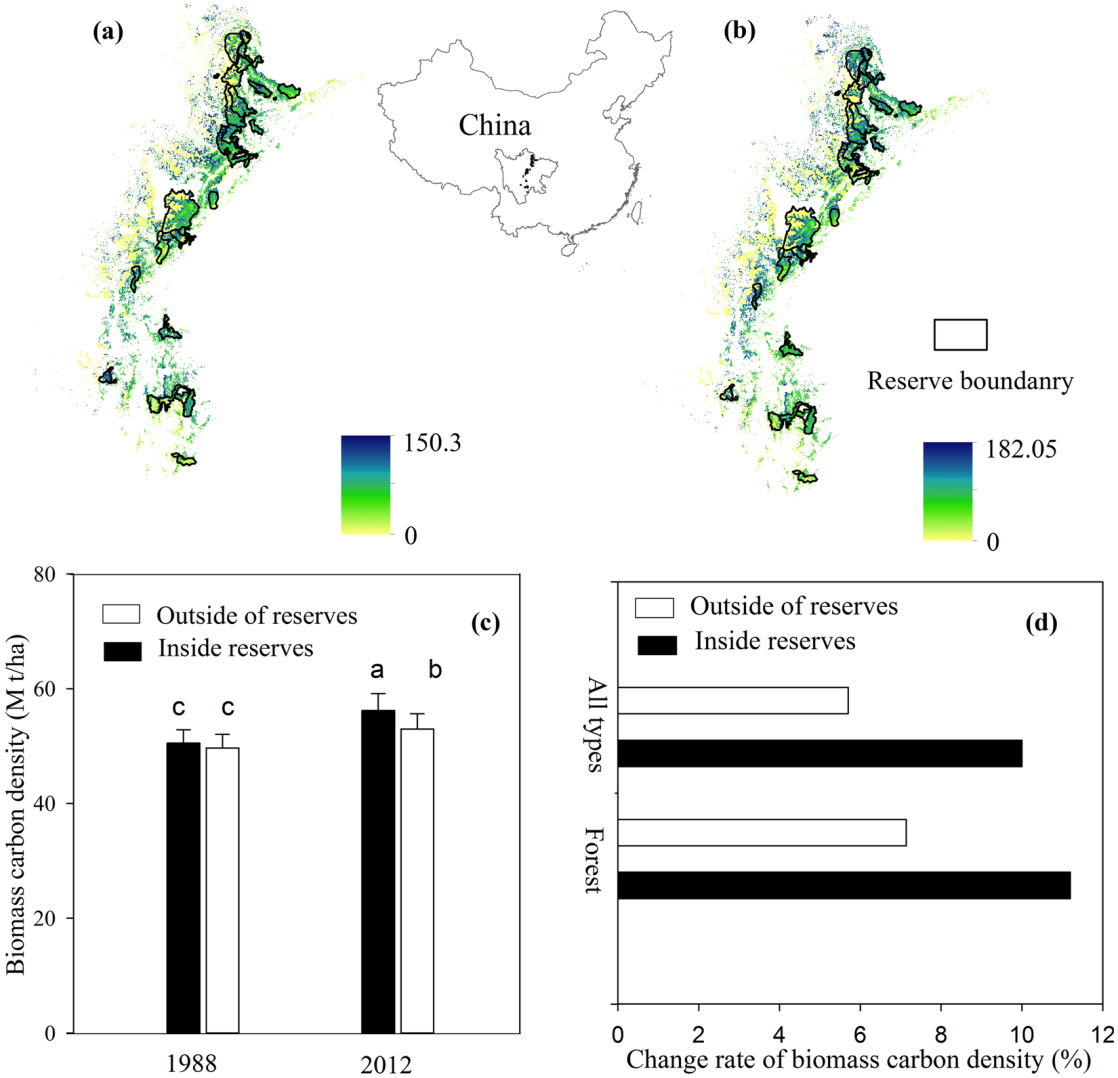
Spatial variation of biomass carbon density (t C ha^−1^) in giant panda reserves and the matched sites outside of the reserves in (**a**) 1988 and (**b**) 2012. (**c**) Pairwise comparisons of mean biomass carbon density inside and outside the reserves (Error bars represent standard errors). (**d**) Relative change rate of mean biomass carbon density between 1988 and 2012 inside and outside of nature reserves for the lands covered by forest and all land types. Maps were made with ArcGIS version 10.2.2 for desktop (http://www.esri.com/software/arcgis).

NRs contributed to improved regional carbon sequestration. The studied NRs and their matched sites (non-reserve; similar land characteristics) contained approximately 56.1 M t C and 55.8 M t C of biomass carbon in 1988, respectively. In 2012, the total biomass carbon inside NRs and matched sites increased to 64.3 M t C and 60.7 M t C respectively, suggesting that the establishment of the NRs increased carbon sequestration by 3.6 M t C. Pairwise comparisons revealed that there was no significant difference in 1988 mean biomass carbon density between protected and unprotected sites. In 2012, it was significantly higher inside the NRs than matching non-reserve sites (Fig. 3c). To distinguish carbon gain through forest regeneration, we calculated the mean biomass carbon density of the entire NRs and those areas covered by forest in both periods. The mean carbon density of the whole NRs increased from 50.5t/ha to 56.2 t/ha, exhibiting a 4.2% higher growth rate compared with lands outside of NRs (Fig. 3d). For the areas covered only by forest in both periods, the mean biomass carbon density increased from 70.0 t/ha to 77.8 t/ha inside the reserves (11.2% increase), and an increase from 69.4 to 74.3 t/ha outside the reserves (7.1% increase; Fig. 3d).

**Figure 4:**
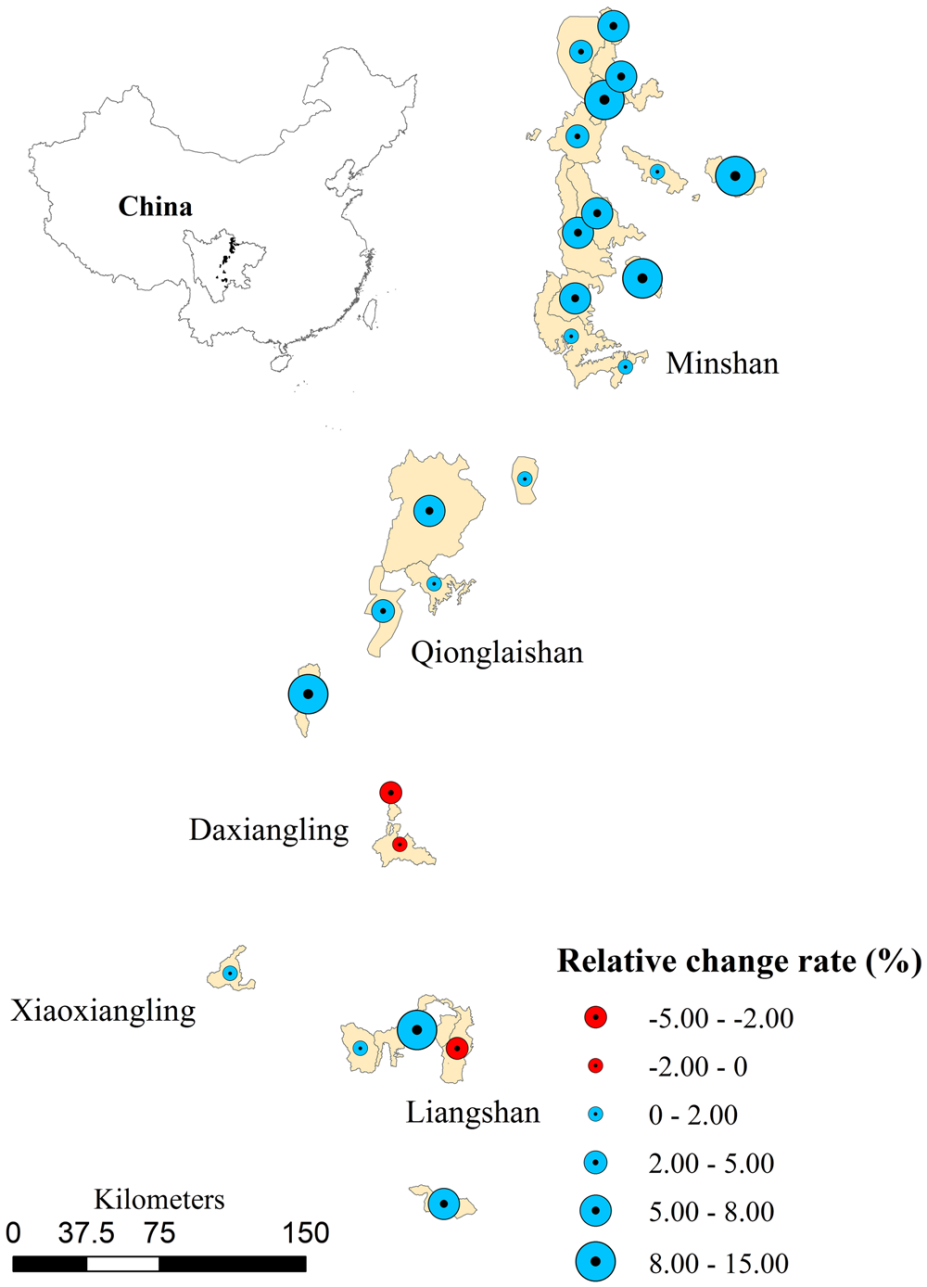
The relative rates of change in mean biomass carbon density in the 25 giant panda nature reserves in five mountain ranges (Minshan, Qionglaishan, Daxiangling, Xiaoxiangling and Liangshan) from 1988 to 2012. Maps were made with ArcGIS version 10.2.2 for desktop (http://www.esri.com/software/arcgis).

We also individually analyzed the contribution of each NR to forest carbon sequestration to illustrate spatial heterogeneity of the impacts. Our results indicated that 23 of 25 NRs showed improved biomass carbon density, with a maximum relative growth rate of 14%. Compared with the matching non-reserve areas, biomass carbon density in 12 NRs increased by over 5%, and 3 NRs (Tangjianghe, Labahe, and Piankou) exhibited a relative growth rate of over 10%. The relative change rate of biomass carbon density in protected sites, compared with non-reserve sites, was found to decrease in only two NRs. These two NRs were located in Liangshan and Daxiangling Mountain Ranges, the southern parts of giant panda habitats in Sichuan Province (Fig. 4).

**Figure 5:**
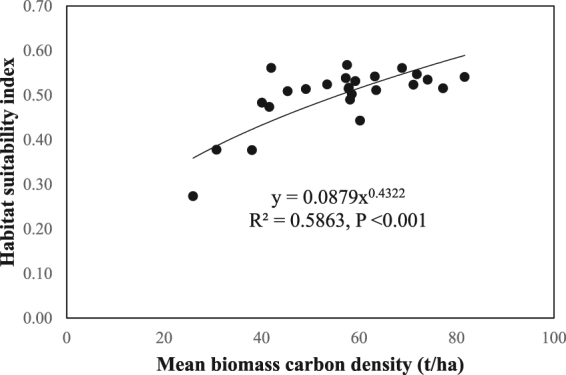
Relationship between mean biomass carbon density and habitat suitability index of giant panda at nature reserve level in 25 panda nature reserves of Sichuan Province. Dotted line indicates a significant relationship.

To investigate potential synergies between carbon stock and giant panda conservation, we explored the relationship between mean biomass carbon density and habitat suitability index at cell scale in each NR and at NR level. We found high congruence between species habitat suitability and biomass carbon density in most of the 25 NRs. Indeed, there were not significant relationships between the two variables in only four NRs (Table 1). We also found that there was a significant correlation between the two variables at NR level (R^2^ = 0.59, p < 0.001) (Fig. 5). Further, the mean carbon density for habitats with different quality was significantly different, i.e. the mean carbon density of the highly suitable habitats was higher than that in the moderately and low suitable habitats. Overall, our results support the expectation that mechanisms for conserving biomass carbon would have substantial co-benefits for giant panda habitat, and vice versa. This to our knowledge for the first time verifies the synergisms between species conservation and carbon stocks at a landscape scale, suggesting the potential co-benefits between species conservation and climate change mitigation in these giant panda habitats.

**Table 1:** Spearman’s rank correlation coefficients (R) between mean biomass carbon density and habitat suitability index in the 25 giant panda nature reserves (^*^P < 0.05, ^**^P < 0.01 and ns = not significant).

Reserve name	R	Reserve name	R
Baihe	0.969^**^	Wolong	0.288^**^
Baicaopo	0.700^**^	Wanglang	0.283^*^
Fentongzai	0.618^**^	Baodinggou	0.274^**^
Anzihe	0.607^**^	Meigudafengding	0.266^**^
Heishuihe	0.597^**^	Xiaozaizigou	0.254^**^
Longxi-hongkou	0.560^**^	Wawushan	0.253^**^
huanglong	0.500^**^	Tangjiahe	0.251^**^
Jiuzaigou	0.405^**^	Xuebaoding	0.214^*^
Qianfoshan	0.356^**^	Yelei	0.117 ns
Piankou	0.336^**^	Shenguozhuang	0.059 ns
Wujiao	0.303^**^	Labahe	0.042 ns
Mabiandafengding	0.298^**^	Baiyang	−0.001 ns
Xiaohegou	0.296^**^

## Discussion

From a social, environmental, resource management perspective, it makes sense that forests should contribute to integrated conservation strategies that address climate change as well as conservation objectives in protected areas^38,39^. Our results indicate that the panda NRs substantially improved biomass carbon density after controlling for terrain, climate, human disturbance and land cover, and support the notion that giant panda conservation can highlight the potential of combining species and carbon conservation in future conservation planning. The benefits of the NRs on improving carbon storage in this study are likely to have been underestimated, because lots of newly established NRs have not been included into our analysis, which may have contributed a lot to carbon stocks in this region. However, our results also indicate that not all NRs perform equally in carbon stock potential. Therefore, to maximize the co-benefits, future conservation measures and funding should focus on primarily those NRs with high quality habitats and high carbon sequestration potential.

There are some feasible ways to achieve both giant panda conservation and climate change mitigation. First, preventing habitat fragmentation and isolation of forest ecosystems in the giant panda NRs would be the best conservation practice. Currently, the wild giant panda population is distributed in more than 30 isolated habitat patches, which can severely reduce gene flow, and population viability^28,30^. Therefore, establishment of ecological corridors and more effective habitat protection that allow for greater mobility among core areas is essential to giant panda conservation in China. Sufficiently increasing the patch size and connectivity of panda habitats may also improve forest carbon stock^40^, in addition to providing additional habitat that could eventually support a source population. A recent study in forest ecosystems found that increasing fragment size has a positive relationship with above-ground carbon stock, indicating that protecting forest fragments, particularly larger fragments, offers important carbon and biodiversity co-benefits in relevant conservation projects^40^.

Second, planting forests is considered the priority management action for carbon sequestration, giant panda habitat restoration and dispersal corridor creation. A mix of trees, including native species, should be planted in potential giant panda habitats, especially in the zones between some isolated patches of giant panda habitats to link them together, since monoculture forests are not considered suitable giant panda habitat, nor are they valuable habitat in general for other wildlife^41–43^. Future conservation efforts should also pay more attention to the forest structure and community composition. It has been suggested that temperate montane broadleaved forest, temperate montane mixed forest, and subalpine forest in this region, with 30–70% canopy cover, provide optimum conditions for bamboo growth and giant panda survival^32^. Using these broad considerations as the basis, it is beneficial to judiciously consider promoting appropriate/optimal forest structure and composition when managing for forests and species conservation.

Third, with giant panda being downgraded, the existing conservation efforts should not be relaxed, but there is a critical need to shift the traditional conservation approach to the new conservation mode. Conservation efforts should sustain the co-benefits of species conservation, carbon sequestration, and other ecosystem services despite the recent downgraded conservation status. The community forest tenure reform surrounding the panda NRs may also potentially threaten panda habitat protection and the carbon co-benefit, especially the high value natural forests^42,43^. Effective eco-compensation could play an increasing important role in restoring giant panda population^42^ and thus enhancing multiple ecosystem services in the future. Alternatively, local communities could take advantage of the carbon co-benefits of panda conservation by selling carbon credits on the domestic and international carbon markets to improve the greatest return on limited conservation investment.

Effective conservation policy will likely require maximizing future opportunities or strategies that concurrently support multiple conservation and management objectives. Such strategies, where possible, could reconcile species conservation and climate change mitigation. NRs, as one of the most successful measures implemented for species conservation contribute to carbon–biodiversity outcomes by improving both carbon sequestration and protecting biodiversity^44–46^. Our results confirm that panda NRs play a critical role in reducing carbon emissions and habitat loss, and reveal important co-benefits between carbon stocks and species conservation in conservation landscapes. This result is consistent with other biodiversity studies, which found a positive spatial relationship between biodiversity and carbon^14,47,48^. This discrepancy between carbon densities is likely attributed to the reduction of logging in forests inside NRs^40^. Such studies show that it is possible to achieve a high level of success in reducing deforestation and carbon emissions through the establishment and implementation of effective conservation policy^49^. Therefore, the setting of regional conservation priorities should receive special attention in the planning investment to foster synergies between carbon and biodiversity, and maximize the potential of species and carbon co-benefits under climate change and limited conservation fund.

## Methods

### Description of the data

We used two plot-based national forest inventory data sets (3615 plots for 1984 to 1988, hereafter referred to as 1988, and 3377 plots for 2008 to 2012, hereafter referred to as 2012) from the Forestry Department of Sichuan Province. The distance between these plots in Sichuan Province is 4 or 8 km. The locations and diameters of all living trees > 5 cm at breast height were recorded. Each recorded tree was numbered and tagged to assist in a subsequent inventory. We also collected stand-based forest inventory data to obtain the spatial distributions and forest origins of 15 species group for 1988 and 2012, covering the 181 counties and districts of Sichuan Province. We carried out field studies in 2010 and 2011 to collect both aboveground (trunks, branches, and leaves) and belowground (roots and stumps) biomass samples for 46 tree species in 167 forest inventory plots, which were randomly selected from all the forest plots in Sichuan Province. Leaves and needles were collected by age class, and woody tissues were collected using a tree increment borer. The C content in each sample was determined with a Vario MAX CN element analyzer (NA Series 2, CE Instruments). The average C content for each species was obtained using the biomass of the different components (leaf, branch, stem, and root) as weighting factors. To build the individual tree-based biomass models, we also harvested 1310 sample trees from different diameter classes in each of the 15 forest species groups for biomass measurements in 2010 and 2011. Details regarding method and procedures can be found in Qiu *et al*. (2015)^50^ and Li *et al*. (2017)^51^.

The digital elevation model data with a resolution of 90 m was provided by International Scientific & Technical Data Mirror Site, Computer Network Information Center, The Chinese Academy of Sciences. The level of human disturbance was expressed based on the distance from residential areas and roads. We obtained a road map (1:250,000) from the National Fundamental Geographic Information Center. We acquired locations of all villages from the Institute of Geographic Sciences and Natural Resources of The Chinese Academy of Sciences. The climate variables for Sichuan Province, including average annual temperature (Tavg) and precipitation (Pre), average annual monthly precipitation (Pre7) and maximum temperature (Tmax) in July, and average annual monthly precipitation (Pre1) and minimum temperature (Tmin) in January were derived from China’s ground-based meteorological data in 1988 and 2012. The land cover maps for 1988 and 2012 derived from Landsat imagery were obtained from the Forestry Department of Sichuan Province, and they were used for identifying forest cover and other land cover types.

### Producing spatially explicit biomass carbon maps

To produce spatially explicit carbon distribution maps in 1988 and 2012, we first calculated plot-based biomass carbon density, and then scaled up the forest biomass carbon in Sichuan Province. The sampling method fully considered the different diameters, ages of forest species, and the distributions of the forest species types. We followed a destructive harvesting method^52^ to measure the aboveground and belowground portions of the biomass (stem, root, branch, and leaf) of each individual tree for the 15 groups of tree species in Sichuan Province.

The individual-tree-based biomass models were established using biomass data of sample trees for 15 groups of species^50^. We derived each tree biomass at each plot by applying the models to individuals from the forest inventory data. The average C contents of the 15 groups of species were used to calculate the tree biomass C. We summarized all the trees into plot levels and converted them to biomass C density per hectare^50^.

Decision-tree modeling with random forest algorithm (RF)^53,54^ was employed for scaling up forest biomass carbon in Sichuan Province, China^50^. We randomly divided forest inventory plots into training data (70%) and testing data (30%). The predictor variables input to RF included tree species distribution, geographic coordinates (X and Y), topographical factors (the slope and aspect), human disturbance (distance to villages and roads), and climate variables. We used the widely adopted thin-plate splines method of ANUSPLIN to interpolate the weather station data and obtain spatial distribution of these climate variables. The R^2^ values between observation and prediction through an independent validation dataset were 0.95 to 0.98 for temperature and 0.8 to 0.85 for precipitation^50^. All analyses were implemented with the R package “Random Forest”. The land cover maps for 1988 and 2012 were used for identifying forest cover and other land cover types. We adopted carbon density data for other land cover types from the Forest Carbon Monitoring and Accounting Project, which was produced by massive field data and land cover data based on IPCC Tier 2 carbon budget assessment method.

### Assessing NR effectiveness of carbon sequestration

In order to avoid the effect of the newly established NRs on the assessment results, we excluded these NRs established in the last 10 years, and finally included 25 NRs in Sichuan Province into our analyses. We compared protected and non-reserve lands using the matching approach^55,56^ to identify the difference of biomass carbon flux and stock between those areas inside and outside NRs since they were not randomly distributed over the landscape. Matching approach is a treatment or policy evaluation method that can help to reduce the influence of the non-random application of a ‘treatment’ (here, nature reserve). For each treated location, we chose the single untreated location that was the most similar to it in terms of the multi-variate distance between the locations’ vectors of land characteristics (tree species distributions, land cover, elevation, slope, distances to roads and villages, climate variables and soil organic carbon) using the Mahalanobis distance specified nearest neighbor matching approach. We carried out all further analyses in R, using the ‘Matching’ package. Summary statistics for all the above variables in controlled, treated and matched site are shown in Table 2. We assessed the differences between protected and unprotected site before and after matching, and found that matched methods greatly improved the similarity of these variables between inside and outside the NRs (Table 2).

**Table 2:** Descriptive statistics features of independent variables before and after matching in treat, control and match sites in panda reserves and outside of these reserves.

Variables	Treat	Match	Control	Treat	Match	Control
	Mean			Sd		
Annual temperature (°C)	4.2	4.3	7.9	4.5	4.5	6.2
Average temperature in January (°C)	−4.9	−4.8	−1.6	4.4	4.5	6.0
Average temperature in July (°C)	12.4	12.5	16.2	4.7	4.7	6.4
Annual precipitation (mm)	81.4	79.5	77.6	13.4	13.7	17.7
Annual precipitation in January (mm)	6.7	6.4	6.5	2.0	2.0	3.2
Annual precipitation in July (mm)	192.7	190.6	194.0	43.4	41.8	55.9
Elevation (m)	3389.6	3368.8	2699.3	939.1	946.2	1334.9
Slop (°)	27.9	27.5	22.8	6.7	6.2	9.3
Distance to village (m)	4903.9	4773.9	2853.7	3602.1	2867.1	2643.1
Distance to main roads (m)	2026.2	2051.0	1239.2	1902.2	1833.5	1325.2
Soil organic carbon (g.kg^−1^)	4.3	4.4	3.4	2.9	2.7	2.9
X coordinate (km)	−191.2	−210.6	−217.6	94.0	111.2	145.6
Y coordinate (km)	3270.0	3267.6	3249.7	166.1	167.1	174.2

We measured the effectiveness of NR in carbon stocks by calculating the amounts and rates of change in forest area and biomass carbon density inside and outside the NRs in 1988 and 2012. We measured the relative change rate in biomass carbon density for individual NR or for the NR network as a whole. We then performed pairwise comparison to identify the difference of carbon density between inside and outside NRs in 1988 and 2012.

### Estimating relationship between habitat suitability and carbon stocks

The maximum entropy approach^57–59^ was employed to simulate habitat suitability for giant pandas^36^. We first used eight bioclimatic variables to model the current distribution probability of 16 bamboo species^37^, and then we built the distribution model for the giant panda using bamboo suitability, the selected eight bioclimatic variables, and five environmental variables (slope, aspect, distance from residential areas, distance from roads, and land cover) as predictors. We adopted a habitat suitability technique to identify the distributions of different classes of habitat suitability for giant pandas. The habitat suitability mode was constructed based on giant pandas’ habitat selection criteria, including bamboo suitability, land cover, elevation, slope, aspect, distance from residential areas, and distance from roads^27,32^. Finally, we reclassified panda habitats into marginally, moderately and highly suitable habitats using standard deviations classification^32^. The details on the simulation of giant panda habitats and bamboo suitability can be found in two papers^36,37^. To investigate the congruence between carbon and biodiversity conservation, Spearman’s rank correlation coefficients were also calculated for the relationships between mean carbon density and habitat suitability index at cell scale and in the whole NR network.
